# Beyond bile acids synthesis: metabolomics profiling highlights extensive metabolic dysregulation and treatment response in CTX

**DOI:** 10.1186/s13023-026-04203-x

**Published:** 2026-01-30

**Authors:** Monte A. Del Monte, Jennifer Hanson, Penelope E. Bonnen

**Affiliations:** 1 Department of Ophthalmology and Visual Sciences, Kellogg Eye Center, University of Michigan, Ann Arbor, MI USA; 2 Department of Molecular and Human Genetics, Baylor College of Medicine, Houston, TX USA

**Keywords:** Cerebrotendinous xanthomatosis, CYP27A1, Metabolomics, Chenodeoxycholic acid, Bile acid metabolism, Phosphatidylethanolamine, Ferroptosis

## Abstract

**Background:**

Cerebrotendinous xanthomatosis (CTX) is an inherited metabolic disorder caused by variants in *CYP27A1* leading to loss of sterol-27-hydroxylase activity. Sterol-27-hydroxylase generates two classes of bioactive signaling molecules: bile acids and oxysterols. The broader metabolic consequences resulting from perturbations in bile acid and oxysterol signaling and their reversibility with FDA-approved treatment chenodeoxycholic acid (CDCA), are not fully described.

**Methods:**

To establish a comprehensive map of metabolic consequences of CTX, we performed large-scale, untargeted plasma metabolomics in a single subject with CTX, both before and after 6 months of CDCA therapy, and compared results with a reference cohort of over 1100 individuals. Data were analyzed for significant metabolite changes and pathway alterations.

**Results:**

Untreated CTX exhibited marked depletion of bile acid intermediates and elevations in sterol precursors, consistent with the known enzymatic block in this pathway. Metabolomics highlighted additional pathways affected by bile acid and oxysterol signaling such as fatty acid metabolism, NAD^+^ de novo synthesis, phosphatidylethanolamines, sphingolipids and ferroptosis. Following six months of CDCA therapy, sterol precursors normalized, bile acid intermediates partially recovered, and phosphatidylethanolamines were restored toward reference ranges, while steroid and phosphatidylcholine metabolites remained largely unchanged.

**Conclusions:**

This study exposes the comprehensive nature of metabolic disturbance in CTX beyond the bile acids pathway, revealing perturbations in bile acids, steroids, fatty acids, phospholipids and NAD+ synthesis and highlights the dynamic early response to CDCA therapy. The metabolomic profile of untreated CTX can be leveraged for diagnostic screening. These findings report new candidate biomarkers for diagnosis and monitoring and underscore the potential of metabolomics to uncover broader metabolic consequences in rare disease.

## Introduction

Cerebrotendinous xanthomatosis (CTX) [OMIM 213700] is a rare inborn error of metabolism (IEM) caused by bi-allelic pathogenic variants in the gene *CYP27A1*, which encodes for the mitochondrial enzyme sterol-27-hydroxylase. Symptoms often begin in the first decade of life with diarrhea, developmental delay, or intellectual disability. Juvenile-onset cataracts, tendon xanthomas, cerebellar dysfunction, pyramidal dysfunction, peripheral neuropathy are common and neurodegeneration progresses as patients age [1–3].

Sterol-27-hydroxylase is a key enzyme in bile acids synthesis. Bile acid synthesis catabolizes cholesterol and yields bile acids cholic acid (CA) and chenodeoxycholic acid (CDCA). Loss of sterol-27-hydroxylase activity leads to accumulation of metabolites upstream of this enzyme including cholestanol, elevation of which is the most widely used diagnostic biomarker for CTX. Conversely, in CTX the production of metabolites downstream of *CYP27A1* in the bile acids pathway are reduced including bile acids. Clinical studies show that FDA-approved treatment CDCA successfully slows CTX disease progression, particularly when initiated at younger ages [4–6].

Two classes of bioactive compounds are produced by sterol-27-hydroxylase activity in the bile acid synthesis pathway: bile acids and oxysterols. In addition to their detergent functions, bile acids function as signaling hormones by serving as ligands for multiple nuclear receptors including the farnesoid X receptor (FXR). Primary bile acid CDCA is the most potent activator of FXR, the activation of which regulates bile acids homeostasis by promoting cholesterol efflux and primary bile acids conjugation while repressing bile acid synthesis, providing a negative feedback loop [7]. Bile acids activation of FXR also promotes adipogenesis, induces lipoprotein clearance, reduces hepatic lipogenesis and gluconeogenesis, and represses ferroptosis [8–11].

Oxysterols, 25-hydroxysterol (25-OHC), 26-hydroxysterol (26-OHC), 27-hydroxysterol (27-OHC) are direct products of sterol-27-hydroxylase enzymatic activity [12]. These intermediate products of bile acid synthesis also function as signaling molecules and are agonists of the nuclear receptor liver X receptor (LXR) receptor. LXR activation promotes lipogenesis, fatty acid oxidation and glycolytic flux while downregulating gluconeogenesis [13, 14]. In the brain, LXR activation by 25-OHC is essential for cerebellar myelination and remyelination in mice [15]. Loss of sterol-27-hydroxylase activity and the resultant deficiency of oxysterols and bile acids is therefore expected to disrupt these interconnected signaling networks and metabolic processes.

Untargeted metabolomics provides a powerful approach to simultaneously interrogate hundreds of small molecules. We conducted large-scale, untargeted metabolomics on the plasma of a subject diagnosed with CTX, both before and after six months of treatment with CDCA. These data recapitulated previous findings of biochemical disturbances in the bile acids synthesis pathway that result from CTX validating this approach to detect changes that accurately reflect this disease state. Metabolomics illuminated disturbances in additional pathways regulated by bile acid and oxysterol signaling that are under-appreciated as being part of the CTX pathomechanism: NAD^+^ synthesis, phosphatidylethanolamine homeostasis and ferroptosis, and sphingolipid S1P. This untargeted approach highlights the comprehensive nature of the metabolic perturbations in CTX and lays the groundwork for a metabolomic profile of CTX that can be utilized for diagnostic screening, future biomarker development and mechanistic investigation.

## Methods

### Ethics statement

All subjects and their families were consented to an Institutional Review Board (IRB)-approved protocol for participation in research study. Clinical data and plasma were collected and published with consent from the families in accordance with consent forms and study protocols approved by IRB of Baylor College of Medicine.

### Genetic Testing

PCR amplification and DNA sequencing by Sanger dideoxy terminators. Both strands of all coding exons and exon/intron boundaries were sequenced. All variants are reported using reference sequence NM_000784.4(CYP27A1).

### Metabolomics

All metabolomics data generation, compound identification, normalization, and quality control procedures were performed by Metabolon, Inc, (Durham, North Carolina) as previously described [16–18]. Plasma samples were processed by Metabolon using a methanol-based extraction protocol to remove proteins and recover small-molecule metabolites. Extracts were prepared according to Metabolon’s (Durham, NC, USA) standard methanol-based extraction protocol [17]. Metabolomic profiling was conducted using Metabolon’s ultra-high-performance liquid chromatography-tandem mass spectrometry (UHPLC-MS/MS) platform, as previously described [17]. Extracted samples were split into equal parts for analysis under complementary chromatographic and mass spectrometric conditions, including reverse-phase and hydrophilic interaction chromatography, with both positive and negative ionization modes. Metabolon’s in-house proprietary software was used to identify metabolites by comparison to Metabolon’s in-house reference library of authenticated standards using retention time, mass-to-charge ratio, and tandem mass spectrometry (MS/MS) spectral data [17, 18]. Metabolon’s in-house proprietary software was also used for quantification of peaks using area-under-the-curve and for data normalization to correct variation resulting from system artifacts, instrument differences and background signal [18]. Metabolite abundances were normalized by Metabolon and expressed relative to a large internal reference population (*n* > 1,100) analyzed using the same UHPLC–MS/MS platform and data processing pipeline. For each metabolite, Z-scores were calculated by Metabolon based on the mean and standard deviation of the reference population distribution, enabling identification of metabolites that were elevated or diminished relative to a broad population baseline [17]. The reference population is not disease-matched and serves as a comparative framework for relative quantification rather than as a control cohort. As the assay is semi-quantitative, reported values represent relative abundances rather than absolute concentrations, and results are interpreted at the level of directional changes and pathway-level trends, consistent with prior applications of this platform [16, 17, 18].

### Statistical analyses

Statistical analyses were conducted to determine if groups of metabolites were different before and after treatment using a two-tailed, unpaired Student’s t-Test.

## Results

### Clinical case description

We describe a previously unreported subject with CTX. The subject was born to a family of Eastern European/Polish ancestry with no history of metabolic disease. He suffered an *in utero*/neonatal stroke-like episode with resulting seizures and chronic non-progressive right hemiparesis. He was also diagnosed with Von Willebrand Disease. He experienced initial constipation and then chronic diarrhea beginning at 3 years of age with soft unformed stools and some urgency, that later resolved with lactose avoidance and CDCA treatment. He developed an acquired exotropia at 7 years of age status post bilateral lateral rectus recession and was diagnosed with idiopathic bilateral rapidly progressive juvenile posterior sub-capsular cataracts at age 11 which required cataract extraction. Neurological abnormalities progressed to include developmental delay, cognitive decline, abnormal gait and balance, hand shaking, and involuntary mouth movements by 16 years of age. He was also noted to have frequent bone fractures throughout childhood.

Biochemical testing in serum showed elevated cholestanol at 3.2 mg/dL (nl ≤ 1 mg/dL). Genetic testing revealed the subject was compound heterozygous for a nonsense variant *CYP27A1*:c.1180_1181delCT;p.(Leu394Alafs*18) and a previously reported pathogenic missense variant *CYP27A1*:c.379C > T;p.Arg127Trp.

### Metabolomics profile of untreated CTX

**Fig. 1:**
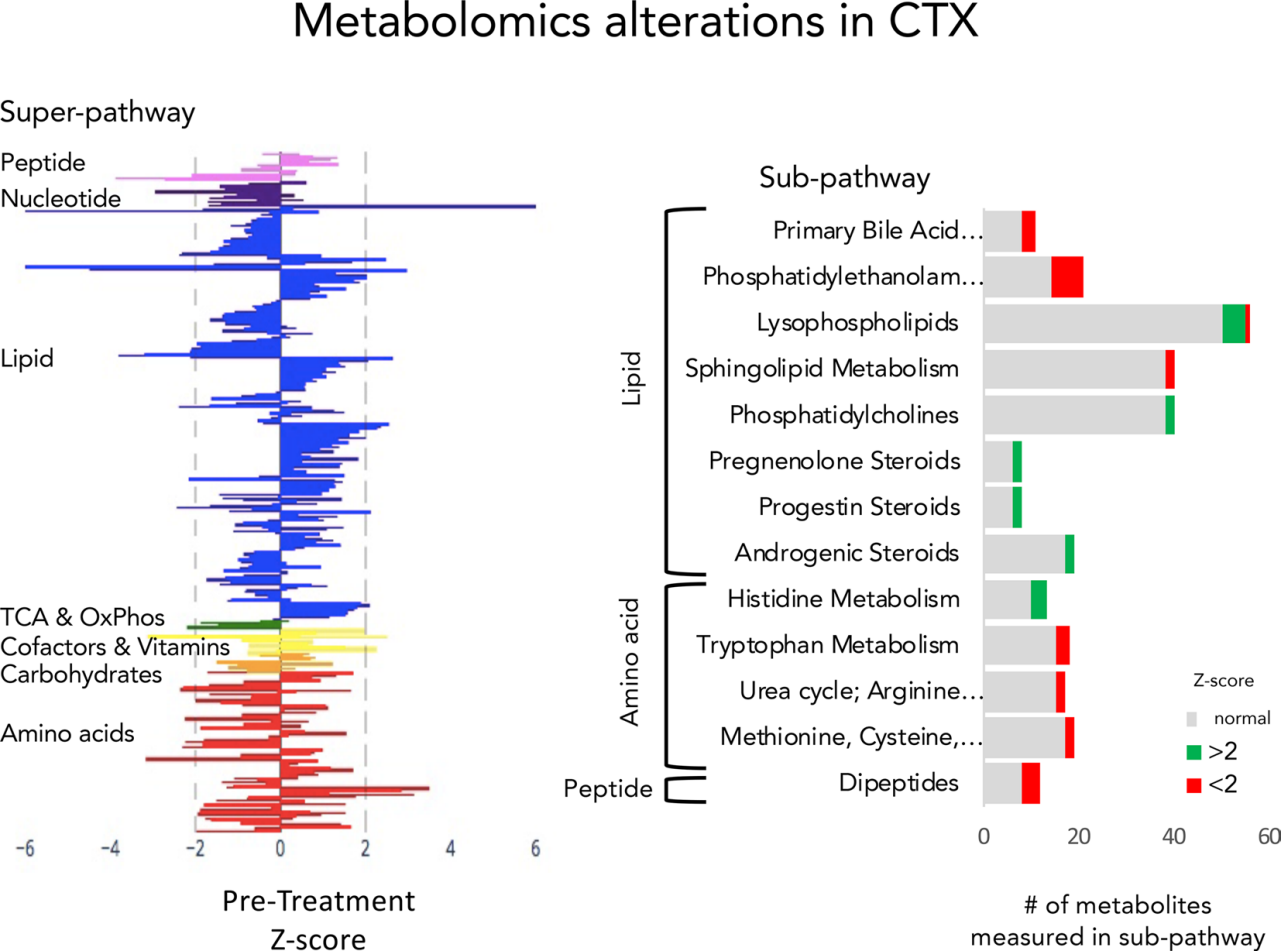
Metabolomics profile in CTX. A. Each bar represents the measurement of an individual metabolite in a single subject who was diagnosed with CTX and sampled prior to beginning CDCA treatment. Metabolites are categorized into super-pathways. Peptides are lavender. Nucleotides are purple. Lipids are blue. TCA and oxidative phosphorylation (OxPhos) are green. Cofactors and vitamins are yellow. Carbohydrates are orange. Amino acids and their metabolic pathways are red. B. The number of metabolites measured in each of the sub-pathways expected to show alterations in CTX. The number of metabolites in each sub-pathway that was elevated relative to control (Z-score > 2) is shown in green and the number of metabolites in each sub-pathway that was diminished (Z-score < 2) is shown in red

Untargeted metabolomics was conducted on plasma of the subject at 15 years of age, prior to initiation of treatment with CDCA. The major pathways that were assessed in this assay were: lipid, peptide, nucleotide, Kreb’s cycle & oxidative phosphorylation, cofactors & vitamins, carbohydrates, and amino acids. Pathways that were most perturbed by CTX were: lipid, peptide, nucleotide, cofactors & vitamins and amino acids (Fig. 1). Lipid metabolites showed the largest magnitude perturbations. Abnormalities were observed across multiple types of lipids: bile acids, sterols, steroids, phosphatidylethanolamines.

### Untargeted metabolomics recapitulates known biochemical abnormalities of CTX validating the use of metabolomics for CTX screening

**Fig. 2:**
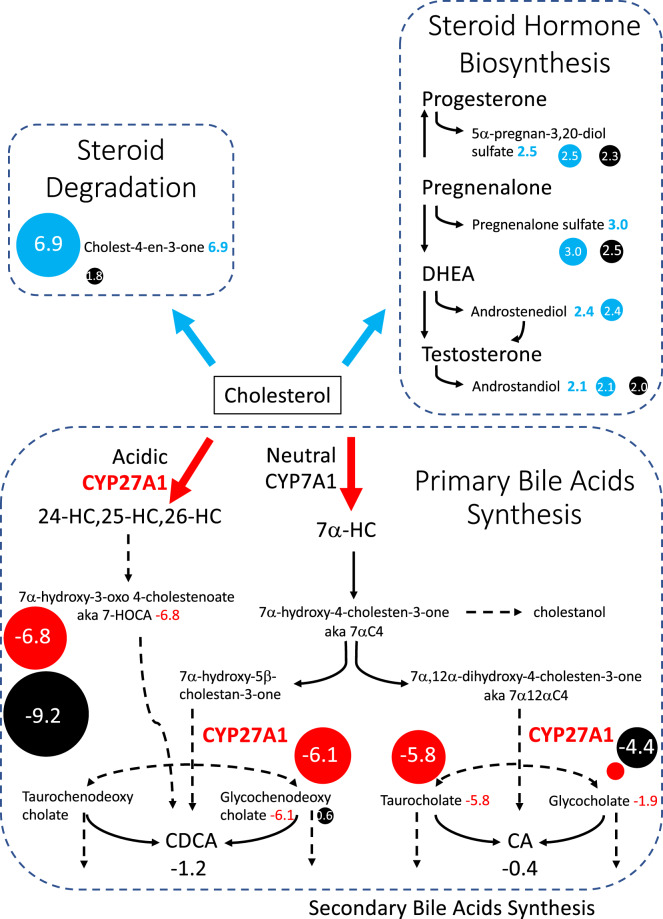
Metabolomics ascertains CTX perturbations in bile acids metabolism and CDCA treatment response. Red circles indicate a metabolite with a value below the range of normal. Blue circles indicate a metabolite with a value above the range of normal. Black circles indicate metabolite values post six months of treatment with CDCA

Bile acids and their intermediates were diminished (Figs. 1, 2). The main pathway for bile acid synthesis involves the conversion of cholesterol into cholic acid (CA) and chenodeoxycholic acid (CDCA). These two bile acids appeared normal: CA (Z-score −0.4) and CDCA (Z-score −1.2). However, 7alpha-hydroxy-3-oxo-4-cholestenoic acid (7-HOCA) was amongst the most perturbed metabolites in the assay and was significantly diminished (Z-score −6.8). 7-HOCA is an intermediate in the acidic bile acids pathway that is produced by the enzymatic step after sterol 27-hydroxylase and is indicator of bile acid synthesis rate. Conjugated bile acids glycochenodeoxycholate (Z-score −6.1) and taurocholate (Z-score −5.8) were among the lowest metabolites measures. Two dipeptides that are products of the bile acids synthesis pathway were also abnormally low: isoleucylalanine (Z-score −2.7) and valylglycine (Z-score −3.9) (Fig. 2).

Sterols and steroid lipids were elevated (Figs. 1, 2). The sterol 4-cholesten-3-one, which is a direct metabolite of cholesterol in the steroid degradation pathway was significantly elevated (Z-score 6.9) (Fig. 2). Pregnenalone, a steroid generated directly from cholesterol, was mildly elevated (Z-score = 3.0) as was progestin (Z-score = 2.5). Sterols beta-sitosterol, campesterol and cholesterol were normal.

### Untargeted metabolomics expands our knowledge of metabolic disturbances in CTX

Phosphatidylethanolamines (PE) were low. Half the phosphatidylethanolamines measured (*N* = 14) were abnormally low and the other half were low end of normal. Polyunsaturated fatty acid, medium chain fatty acids, long chain fatty acids, acyl carnitines were normal.

**Fig. 3:**
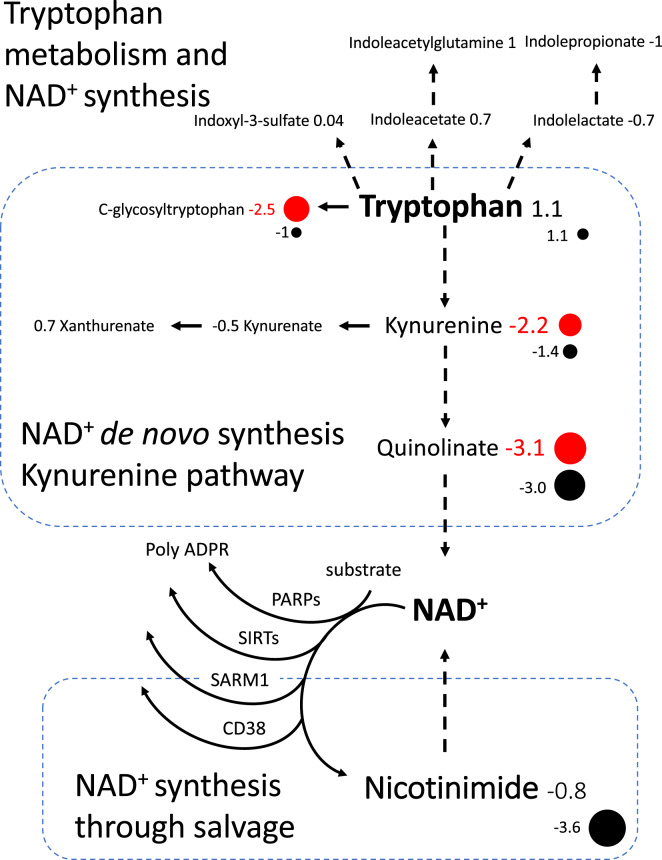
Tryptophan metabolism and NAD^+^ synthesis were diminished in CTX and modulated by CDCA treatment. Red circles indicate a metabolite with a value below the range of normal. Black circles indicate metabolite values post six months of treatment with CDCA

NAD^+^ (nicotinamide adenine dinucleotide) de novo synthesis pathway was disrupted. NAD^+^ is produced de novo via catabolism of tryptophan through the kynurenine pathway. While tryptophan levels were normal (Z-score 1.2), its metabolites in the NAD^+^ de novo synthesis pathway were diminished: quinolate (Z-score −3.1), kynurenine (Z-score −2.2), C-glycosyltryptophan (Z-score −2.3) (Fig. 3). Quinolate (quinolinic acid) is an NMDA receptor agonist that increases glutamate release and promotes excitatory neurotransmission. Kynurenate (kynurenic acid) which is a direct metabolite of kynurenine helps protect neurons from overexcitation and is an antagonist of glutamatergic and cholinergic receptors as well as modulates extracellular levels of glutamate, acetylcholine, and GABA [19].

### Metabolomics reflected known effects of CDCA treatment on bile acid metabolism and steroid degradation

Six months after initiation of treatment with CDCA, plasma was sampled and untargeted metabolomics was conducted using the same assay as before treatment. Treatment in CTX patients with physiological and supra-physiologic levels of CDCA has two main effects. First, CDCA acts as a replacement therapy by providing the patient with normal levels of the primary bile acid CDCA, which is low or absent in these patients. Additionally, the presence of ample CDCA triggers inhibition of bile acids synthesis through the negative feedback loop that regulates this pathway. This leads to normalization of intermediates proximal to CYP27A1 in the bile acids synthesis pathway that had built up due to the blockage resulting from loss of function variants in CYP27A1. This downregulation of the bile acids pathway also results in further decreases in the metabolites that are distal to CYP27A1 in the pathway.

Metabolomics reflected these effects of CDCA treatment on bile acid metabolism. Individuals metabolites experienced significant change in value when exposed to CDCA treatment. 7-HOCA was low before treatment and decreased further post treatment (Z-score −6.8 to −9.2) reflecting the known downregulation of bile acids synthesis (Fig. 2). Similarly, glycocholate decreased to (Z-score −1.9 to −4.4). Two glycosylated forms of CDCA were abnormally low prior to treatment and normalized with CDCA treatment (glycochenodeoxycholate (Z-score −6.1 to 0.6) and glycochenodeoxycholate sulfate (Z-score −4.5 to 0.2)) reflecting glycosylation of supplemented CDCA.

Some individual sterols and steroid lipids were elevated prior to treatment with CDCA and all of them decreased toward normal on treatment. The sterol 4-cholesten-3-one, which is a direct metabolite of cholesterol, was significantly elevated (Z-score 6.9) and completely normalized with treatment (Fig. 2). Androgenic steroids increased overall (*N* = 16, p-value = 0.002) with four increasing to slightly above normal. Pregnenolone steroids did not appear to experience significant change (*N* = 5, p-value = 0.6) and pregnenolone sulfate decreased in response to treatment but remained elevated (Z-score 3.0 to 2.5). Progestin steroids pregnanolone and 5alpha-pregnan-3,20-diol sulfate diminished but remained mildly elevated.

### Metabolomics of CTX while on CDCA treatment revealed broad effects of modulation of bile acids

Metabolomic profiling of CTX on CDCA therapy revealed broad metabolic effects beyond the bile acids synthesis pathway. These findings reflect the wide-ranging effects of the bioactive signaling molecules generated by bile acids synthesis: bile acids and oxysterols.

### Fatty acid metabolism was decreased by CDCA

**Fig. 4:**
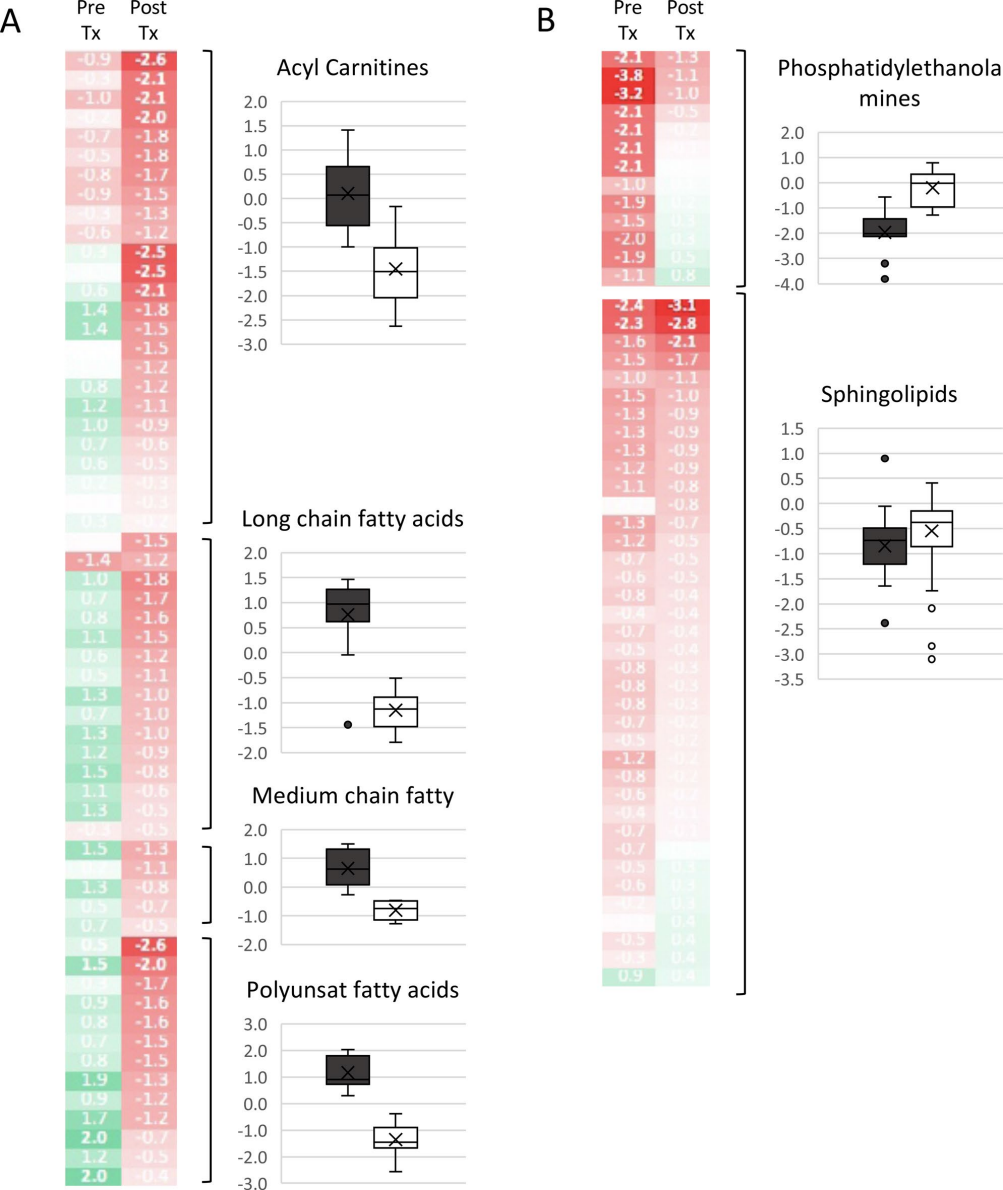
CDCA treatment in CTX lowers fatty acids and increases phosphatidylethanolamines. Untargeted metabolomics in plasma of a subject with CTX pre-treatment (Pre Tx) and after six months of treatment (Post Tx) with CDCA. Acyl carnitines and fatty acids were decreased after treatment. Box and whiskers plots for each group of metabolites before and after treatment is shown. Green indicates metabolites that had Z-score > 0. Red indicates metabolites that had Z-score < 0. Color intensity deepens as number move away from zero

FXR activation inhibits fatty acid synthesis through reducing expression of fatty acid synthesis genes [9]. As the major ligand of FXR, CDCA, triggers this inhibition resulting in decreased fatty acid metabolism. Fatty Acid Metabolism (Acyl Carnitine) (*N* = 25, p-value 2.5E-9) all were normal before treatment and they all decreased on treatment (Fig. 4). Seven became abnormally low with treatment. Long Chain Fatty Acid (*N* = 15, p-value 5.7E-8) all were normal before and during treatment and they all decreased on treatment (Fig. 4). Medium Chain Fatty Acid (*N* = 6, p-value 9.4E-3) all were normal before and during treatment and they all decreased on treatment (Fig. 4). Polyunsaturated fatty acids (*N* = 13, p-value 6E-10) all were normal before treatment and all decreased with treatment (Fig. 4). One decreased below normal range.

Sphingolipid metabolism also changed in response to CDCA (*N* = 38, p-value 1.8E-4) (Fig. 4)). Sphingosine-1-phosphate Receptor 2 (S1PR2) is a non-canonical bile acids receptor that induces the conversion sphingosine to sphingosine-1-phosphate (S1P) and elevates expression of genes associated with hepatic glucose and lipid metabolism [20]. In our subject before treatment sphingosine 1-phosphate was low (Z-score −2.4) and it decreased (Z-score −3.1) on treatment. Sphingosine was in the normal range pre-treatment and lowered below normal after treatment. Sphingomyelins (*N* = 33) were all normal before and after treatment and they all increased with treatment.

### Phosphatidylethanolamines were favorably modulated by CDCA treatment

All phosphatidylethanolamines (PE) assayed (*n* = 14) increased on CDCA treatment (p-value 1.3E-6) (Fig. 4). Seven PE were abnormally low and returned to the normal range on treatment. Others were low end of normal and increased but remained within the normal range. The three most depleted PEs contained arachidonic acid: 1-stearoyl-2-arachidonoyl- GPE (Z-score −3.8), 1-palmitoyl-2-arachidonoyl-GPE (Z-score −3.2), 1-oleoyl-2-arachidonoyl-GPE (Z-score −2.1).

In the liver, PE can be converted to phosphatidylcholine (PC). Phosphatidylcholine (PC) (*n* = 38) (p-value 2.4E-6). All but 6 decreased in value. Prior to treatment two PC were elevated and decreased to normal range on treatment. All others were normal before and after treatment. PEs that contain arachidonic acid and adrenic acid are essential to ferroptosis death signaling [21]. Ferroptosis, a form of regulated cell death that is iron-dependent and is mediated by lipid peroxidation, has been shown to be inhibited by FXR activation [9].

### NAD^*+*^ de novo synthesis and salvage remain abnormal in early treatment

Metabolites in the NAD^+^ de novo synthesis pathway were diminished before treatment. Some of these appeared to normalize, however, quinolate (Z-score −3.1 to −3.0) remained low (Fig. 3). While NAD^+^ can be produced de novo via catabolism of tryptophan, most NAD^+^ in the body is recycled from nicotinamide (Fig. 3). This salvage pathway generates NAD^+^ from nicotinamide generated through NAD^+^ consuming reactions of SIRTs, PARPs, CD38, and SARM1 [22] (Fig. 3). Nicotinimide went from normal levels pre-treatment to significantly low levels with CDCA treatment (Z-score −0.8 to −3.6). The NAD^+^ dependent enzymes in the NAD^+^ salvage pathway also interact with FXR and/or LXR and the depletion of nicotinamide may result from FXR activation by CDCA.

## Discussion

This study provides a comprehensive untargeted metabolomics profile of a patient with cerebrotendinous xanthomatosis (CTX), both before and after six months of chenodeoxycholic acid (CDCA) therapy. Our findings not only recapitulate known metabolic perturbations in CTX, such as disrupted bile acid synthesis and elevated cholesterol precursors, but also reveal novel insights into downstream metabolic consequences effected through oxysterol and bile acids signaling. These results demonstrate the broad systemic impact of sterol-27-hydroxylase deficiency and highlight the utility of metabolomics for biomarker discovery and greater mechanistic understanding.

Metabolomics analysis of CTX, pre-treatment, reflected disruption in the bile acids synthesis pathway. The observed reductions in bile acids and bile acid intermediates downstream of CYP27A1 (e.g., 7alpha-hydroxy-3-oxo-4-cholestenoate [7-HOCA], glycochenodeoxycholate) and elevations in sterol intermediates upstream (e.g., 4-cholesten-3-one) reflect impaired flux through the bile acid pathway and accumulation of upstream metabolites. Elevated steroid metabolites, including pregnenolone and progestin derivatives, reflect increased substrate availability due to impaired cholesterol catabolism through the bile acids synthesis pathway. CDCA treatment normalized 4-cholesten-3-one, other steroid classes showed minimal change, suggesting partial but incomplete restoration of steroid homeostasis within the six-month timeframe of this study. Bile acids intermediates distal to CYP27A1 (7-HOCA) were decreased even lower by CDCA treatment as a result of the negative feedback supplemental CDCA triggers. These results recapitulate previous findings on biochemical disturbances in the bile acids synthesis pathway that result from CTX and CDCA treatment and serve as validation of this untargeted metabolomics approach for accurately discerning changes that represent alterations in CTX and CDCA treatment.

Fatty acids were normal in CTX pretreatment and they were all diminished on CDCA treatment. Most stayed within the normal range, however, seven acyl carnitines dropped lower than the normal range. This decrease in fatty acids is in line with the finding that FXR activation inhibits fatty acid synthesis through reducing expression of fatty acid synthesis genes [9]. It is not currently common practice to monitor acyl carnitine levels during treatment with CDCA and this could potentially be something to explore.

Sphingolipids were also modulated by CDCA treatment. Previous studies showed CDCA increases the activity of sphingosine kinase, the enzyme that converts sphingosine to S1P and activates S1PR2 [20, 23]. Sphingosine-1-phosphate Receptor 2 (S1PR2) is a non-canonical bile acids receptor that induces the conversion of sphingosine to sphingosine-1-phosphate (S1P) and elevates expression of genes associated with hepatic glucose and lipid metabolism [20]. In our subject, S1P and sphingosine both decreased in response to CDCA. Conversely, all other sphingomyelins (*N* = 33) increased with CDCA treatment and were normal before and after treatment.

There was significant reduction in plasma phosphatidylethanolamines (PE) prior to CDCA therapy, particularly PE species containing arachidonic acid. This underscores findings linking CDCA activated FXR signaling to regulation of ferroptosis, an iron-dependent form of programmed cell death that relies on PE species enriched in arachidonic acid [10, 21]. PEs that contain arachidonic acid and adrenic acid are essential to ferroptosis death signaling [21]. Ferroptosis, a form of regulated cell death that is iron-dependent and is mediated by lipid peroxidation, has been shown to be inhibited by FXR activation [9]. Following CDCA treatment, PE levels were restored toward normal suggesting that bile acid supplementation may modulate ferroptosis susceptibility by restoring PE homeostasis in CTX patients.

NAD^+^ synthesis appeared diminished pre-treatment and post. Prior to treatment the CTX patient showed lower metabolites in the NAD^+^ de novo synthesis pathway but normal nicotinamide. After six months of CDCA treatment quinolate levels remained low (Z-score −3) and nicotinamide decreased significantly. The decrease in nicotinamide could possibly result from increased demand of NAD^+^ resulting from CDCA agonizing FXR activation; these perturbations may normalize after longer treatment but further study is needed.

The precise mechanisms underlying the full clinical presentation of CTX remains to be elucidated. The metabolomics data in this study emphasize the complex signaling conducted by bile acids and oxysterols and points to multiple aspects of disease biology that may contribute to multiple patho-mechanisms underlying this progressive, multisystem disorder. There are rare and common diseases that manifest disruptions in pathways outside of primary bile acids synthesis that show perturbations in this CTX plasma metabolomics data. Rare disease caused by pathogenic variants in genes in oxysterol and phosphatidylethanolamine synthesis pathways typically feature motor neuron disease [24]. Ferroptosis as well as S1P may be involved in more common neurodegenerative diseases including Alzheimer’s disease, Parkinson’s disease, Huntington’s disease, multiple sclerosis, and amyotrophic lateral sclerosis [25]. Further study of these pathways is warranted.

This study design has limitations that warrant discussion for interpretation of these results. This analysis is based on a single individual with CTX, precluding broad generalization and inferences on inter-individual variability. In addition, this untargeted metabolomics assay is semi-quantitative and reports values relative to controls; absolute concentrations and metabolic flux are undetermined. Third, this study is restricted to plasma metabolomics, which reflect systemic metabolic signaling but does not capture tissue-specific or central nervous system metabolism. Instead, these results lay groundwork for development of non-invasive biomarkers for monitoring disease progression and treatment response.

The untargeted, systems-level approach employed here successfully recapitulated known biochemical abnormalities of CTX while also revealing previously under-appreciated pathway perturbations, including phosphatidylethanolamine homeostasis, NAD^+^ metabolism, sphingolipid signaling, and ferroptosis-related lipid biology. These findings highlight the value of deep metabolic profiling in rare disease and provide a foundation for future longitudinal, multi-subject, and tissue-specific investigations aimed at linking systemic metabolic signaling with neurological pathology and treatment response.

## Data Availability

The data presented in this study are available on request from the corresponding author to ensure responsible use and confidentiality.
